# Leveraging AI to Democratize the Hidden Curriculum in Medical Education: An Implementation Framework

**DOI:** 10.1007/s40670-025-02497-3

**Published:** 2025-09-06

**Authors:** James Keith Martin, Mercedes Byrd

**Affiliations:** 1 Department of Medical Education and Scholarship, Rowan-Virtua School of Osteopathic Medicine, 113 Laurel Rd, Stratford, NJ 08084 USA; 2 Rowan-Virtua School of Osteopathic Medicine, Hope Brings Strength Library at Rowan-Virtua SOM, 1474 Tanyard Rd, Sewell, NJ 08080 USA

**Keywords:** Artificial intelligence, Medical education, Hidden curriculum, Educational equity, First-generation students, Cognitive load

## Abstract

The “hidden curriculum” in medical education—comprising unwritten rules, values, and expectations—significantly impacts student success, yet remains inaccessible to students from underrepresented backgrounds. This paper presents a theoretical framework and practical implementation strategy for using artificial intelligence (AI) to democratize access to this hidden curriculum. We analyze how cognitive load theory and the Fast/Slow Thinking paradigm explain inequities in professional integration, then propose a comprehensive implementation approach to guide equitable AI integration. This model demonstrates how AI tools, when thoughtfully implemented, can reduce cognitive burdens on disadvantaged students, accelerate professional acculturation, and contribute to building an inclusive medical workforce.

## Inequitable Access to the Hidden Curriculum

Medical education follows formal competency guidelines, but also contains a crucial “hidden curriculum” of unspoken values, behaviors, and procedures essential for student success [1]. These implicit expectations govern how students navigate medical education from basic science courses through clinical rotations, build effective study strategies, secure research opportunities, optimize academic performance, and strategically position themselves for residency [2]. This knowledge is vital for maximizing educational resources and career opportunities, yet it remains absent from formal instruction.

Information about navigating these implicit systems typically flows through informal channels, including peer networks, generational knowledge, and online forums such as Reddit [3]. This creates a significant disadvantage for students from underrepresented backgrounds, particularly first-generation, low-income (FGLI) students and underrepresented minority (URM) groups [4, 5]. These students often lack access to the networks and generational knowledge that provide the “script” of the hidden curriculum [6]. They experience a “lag phase” in professional integration across all stages of training and face additional challenges that can impede their entry into certain medical specialties [7].

Generative artificial intelligence (GAI) offers a promising mechanism to democratize access to the hidden curriculum by centralizing implicit knowledge, providing 24/7 virtual mentorship, and delivering just-in-time guidance. This can help reduce the integration lag phase experienced by FGLI/URM students. This article presents a novel framework for implementing AI-driven equity interventions in medical education that specifically targets hidden curriculum inequities, offering a scalable approach to transform how FGLI/URM students access and navigate implicit professional knowledge throughout their medical training.

## Multifaceted Challenges Facing Underrepresented Students

FGLI/URM students face complex obstacles beyond basic knowledge gaps. Many might struggle with:

- *Understanding the medical education process*: Recent research confirms that first-generation medical students face unique challenges in navigating medical education systems that their peers with familial or social exposure to medical culture do not encounter [8]. FGLI students tend to take leave of absence at higher rates than their peers [9], indicating the additional burden of understanding complex medical education processes.
- *Financial barriers beyond tuition*: The cost of medical school exceeds $70,000 annually at some private institutions, creating a major financial barrier [10]. Hidden costs, including study resources, board examination fees, professional attire, and away rotations, create additional burdens for economically disadvantaged students [11].
- *Finding community and mentorship*: The absence of physician mentors with similar backgrounds negatively impacts FLGI/URM students, limiting their access to role models and sponsors [11, 12]. A recent study confirms that first-generation medical students feel they face disproportionate adversity and do not receive adequate or targeted institutional support [13].
- *Balancing unfamiliar cultural environments with cultural responsibilities*: Many FGLI/URM students often lack the cultural capital—the knowledge and skills that make educational systems feel comfortable and familiar—necessary for seamless integration into medical culture [6]. These students often have family responsibilities that their more privileged peers do not, creating additional time constraints and stressors throughout both pre-clinical and clinical years [14].

These challenges combine to create a “lag phase” in professional integration, where underrepresented students must simultaneously master medical content while learning to navigate unfamiliar social and professional systems [7]. This additional cognitive burden represents a significant educational inequity rarely acknowledged in formal curriculum design or student support services.

## The Fast/Slow Thinking Paradigm in Educational Equity

To understand the inequitable impact of the hidden curriculum, we applied the Fast/Slow Thinking paradigm. This paradigm distinguishes between two modes of thought [15]:

- *Fast thinking*: automatic, intuitive processing requiring minimal cognitive resources
- *Slow thinking*: deliberate, analytical processing requiring substantial cognitive resources

For students with generational knowledge, many aspects of the hidden curriculum can be processed through fast thinking. The unwritten rules align with their pre-existing mental models, allowing efficient navigation with minimal cognitive load.

In contrast, FGLI/URM students must consciously recognize and analyze each unspoken expectation, consuming cognitive resources that their peers can dedicate to content mastery [16]. Understanding the unwritten hierarchy of clinical teams, professional communication, or implicit expectations for research engagement all require conscious analysis for those without insider knowledge, representing a significant but often unrecognized inequity in medical education.

## AI-Driven Solutions for Fast and Slow Thinking Challenges

AI tools can strategically address both types of cognitive challenges:

### Fast Thinking Tasks

- *Professional language decoder:* AI assistants can create specialized glossaries of medical education jargon and institutional acronyms that FGLI/URM students might not recognize. For example, NotebookLM could maintain a searchable repository of terms like “gunner,” “Step prep,” or institution-specific terminology with contextual explanations of their implications.
- *Cultural navigation guides:* AI tools could provide just-in-time guidance on unspoken professional norms, such as email etiquette or how to appropriately request research opportunities. Claude could offer templates and examples specific to different contexts, e.g., approaching a potential research mentor vs. asking for clinical shadowing.
- *Parallel curriculum planner:* NotebookLM could analyze successful pathways of previous students and generate personalized extracurricular roadmaps showing when most students join interest groups, begin research, or prepare for certain exams—making visible the timeline that students with generational knowledge inherently know.

### Slow Thinking Tasks

- *Strategic study planning:* NotebookLM could analyze successful study approaches from previous students and help FGLI/URM students develop personalized study strategies optimized for their learning style and the institution’s specific curriculum and assessment methods.
- *Medical education journey mapping:* NotebookLM could help students visualize their entire medical education journey with interactive timelines that highlight critical decision points, preparation periods, and strategic opportunities that are rarely made explicit in formal curricula.
- *Career planning guidance:* AI tools could provide personalized residency application strategies and specialty selection frameworks based on a student’s background and goals, aggregating successful approaches typically known only through family connections or experienced mentors.

## Implementation and Equity Mechanisms

### Cognitive Load Theory Application

Cognitive load theory (CLT) offers insights into how technological interventions might address these disparities. CLT distinguishes between intrinsic load (inherent complexity of learning material), extraneous load (cognitive burden unrelated to learning objectives), and germane load (productive cognitive effort leading to schema development) [17].

For FGLI/URM students, navigating the hidden curriculum may impose substantial extraneous cognitive load, diverting mental resources from the germane processing of the formal curriculum. AI can reduce this extraneous load by making implicit expectations explicit.

### AI as an Equity Tool

AI offers a powerful medium to democratize access to the hidden curriculum in medical education [18]. While AI in medical education typically focuses on learning assistance and clinical decision-making [19], well-designed tools can address educational inequities by making implicit expectations explicit through several key mechanisms:

- *Increasing Transparency Through Knowledge Centralization:* Studies highlight how AI tools can serve as knowledge aggregators that compile and organize implicit knowledge from multiple sources into coherent, searchable repositories [20–22]. These systems can document unwritten expectations across different educational phases. By capturing the tacit knowledge traditionally available only to students with existing connections to the medical profession, AI systems may help level the informational playing field.
- *Supplementing Mentorship Through Virtual Guidance - providing “just-in-time” support:* While human mentorship remains invaluable [23], AI can provide on-demand answers, to students who lack extensive networks or feel uncomfortable asking basic questions. AI mentoring offers unique advantages for underrepresented students: 24/7 accessibility without fear of judgment or concern about imposing on busy faculty and a safe space for questions about basic knowledge or cultural norms that others take for granted. Research shows students from underrepresented backgrounds often hesitate to seek clarification about unwritten rules due to impostor syndrome and fear of confirming negative stereotypes [11]. Advanced AI systems provide context-aware guidance tailored to a student’s current educational stage, upcoming milestones, and personal background; that deliver relevant information precisely when needed. For example, AI might recognize when a pre-clinical student approaches critical transitions—such as first standardized patient encounters or clinical rotations—and proactively provide targeted guidance. This just-in-time support is particularly valuable for students lacking generational knowledge that would alert them to important opportunities or potential pitfalls [4]. By accelerating the understanding of unwritten rules and providing timely interventions, AI can reduce the “lag phase” and help prevent the compounded disadvantages that occur when students miss critical early opportunities due to a lack of insider knowledge [7, 14].
- *Facilitating Inclusive Learning Environments:* Beyond individual guidance, AI systems can help create more inclusive learning environments by identifying and addressing systemic barriers in the hidden curriculum. These tools can analyze patterns in student experiences and outcomes to illuminate aspects of the hidden curriculum that disproportionately impact underrepresented students. By collecting and analyzing data on student interactions, performance, and professional development, AI can help institutions identify where implicit biases or structural barriers may be impeding the success of certain student groups. This insight would enable the development and implementation of targeted interventions to modify aspects of the hidden curriculum that perpetuate inequities [24, 25].
- *Integration with Learning Management Systems (LMS):* Integrating AI with the existing LMS tools used in education can significantly enhance its effectiveness as an equity tool. When embedded within the digital platforms that students already use, AI tools can seamlessly deliver personalized guidance and support without requiring additional technological adoption or learning curves. LMS integration allows for contextual awareness, with AI systems able to recognize where students are in their educational journey and deliver appropriate guidance based on their current activities. This integration can also facilitate the collection of non-identifying data to continuously improve the AI’s understanding of common challenges and effective interventions for underrepresented students [26]. Recent implementations have demonstrated how AI-enhanced LMS platforms can support equitable access to the hidden curriculum by embedding guidance about unwritten expectations directly into educational resources [27]. This embedded approach ensures that all students, regardless of background, receive the same foundational knowledge about navigating the medical education system.

### Specific AI Tools and Implementation Strategies

Advanced AI assistants, including Anthropic’s Claude, OpenAI’s ChatGPT, Google’s Gemini, and Microsoft’s Copilot, can be used to deliver personalized guidance tailored to students at every level [18]. Pre-clinical students can benefit from knowledge bases focused on foundational sciences and study techniques, while clinical students gain support for rotation-specific challenges and specialty selection. These systems can facilitate both quick clarification of immediate expectations and in-depth exploration of complex decisions.

However, AI “hallucination”—providing incorrect information without indicating uncertainty—remains a concern in education. While several of the leading AI companies address this through reference features, Google’s NotebookLM offers an alternative approach. NotebookLM was chosen for this framework because it allows users to upload up to 50 validated sources that serve as the exclusive knowledge base for the AI tool, ensuring reliance on verified, institutional content rather than potentially unreliable internet information. This theoretically eliminates the risk of AI-generated misinformation that could mislead students about critical expectations.

A pilot implementation demonstrated this potential when pre-clinical students approached faculty during a challenging block. Faculty assisted in the creation of a NotebookLM workspace with curated sources: library resources, course syllabus, and learning objectives. The AI generated personalized study guides and progress trackers to help students understand exactly what to master. This targeted approach proved effective for participants, suggesting broader applicability for addressing gaps through source-constrained AI guidance.

NotebookLM workspaces might include basic sciences materials and study strategies for pre-clinical students, or specialty guides and residency timelines for clinical students. With its collaborative capabilities and Google Workspace integration, NotebookLM presents a trustworthy solution for addressing the hidden curriculum through AI while maintaining strict adherence to verified institutional knowledge throughout a student’s journey from classroom to clinic.

### Integration Across Medical Education

Moving from theoretical potential to practical implementation, these AI tools require systematic integration across institutional structures to achieve sustained equity outcomes. Effective deployment depends on embedding these technologies within existing support ecosystems while establishing continuous feedback mechanisms with diverse stakeholders, including FGLI/URM students whose experiences must inform ongoing refinement and adaptation.

- *Integration with existing educational frameworks:* Integrating AI into medical education requires careful attention to several factors, with cognitive science principles guiding design decisions. AI interfaces should replicate navigation patterns that privileged students find intuitive, helping FGLI/URM students develop “fast thinking” responses to common scenarios. Direct LMS integration minimizes additional cognitive burden. AI prompts can automatically address the contextual questions privileged students would instinctively ask, such as “What are the unwritten expectations for this rotation?” or “How should I interpret this feedback?” Additionally, AI responses should be structured to minimize intrinsic load by breaking complex hidden curriculum concepts into digestible, sequential steps rather than overwhelming students with comprehensive but cognitively demanding information dumps.
- *Curriculum integration:* AI integration requires alignment with curricular structures and accreditation requirements. Interventions should target “slow thinking” moments for FGLI/URM students—clinical rotations, research opportunities, specialty selection—where privileged peers receive family guidance. Phase-specific modules should match cognitive capacity at each educational stage, ensuring focus on professional development over system navigation. Integration with existing mentorship creates touchpoints for combined AI and human support.
- *Faculty development:* Faculty engagement is critical to successful implementation. Comprehensive development programs should train instructors on equitable AI implementation while avoiding technological barriers that disadvantage certain populations. Clear guidelines must define appropriate tool use and limitations, ensuring faculty understand when AI assistance is beneficial versus when human guidance remains essential. Regular review processes maintain accuracy and relevance, while faculty champions across departments facilitate knowledge sharing and consistent institutional implementation.
- *Ethical frameworks and implementation considerations: *While AI holds significant promise as an equity tool in medical education, implementation requires careful consideration to avoid creating new disadvantages. AI algorithms can contain biases if built on insufficiently diverse datasets [19], and data privacy concerns risk reinforcing existing inequalities [20]. Developing tools with diverse teams and robust ethical frameworks ensures AI promotes rather than perpetuates disparities [21]. Additionally, AI must complement rather than replace critical thinking and problem-solving skill development [12]. The goal is to level the playing field while maintaining educational rigor.
- *Data privacy and security:* AI implementation requires robust data protection through clear governance policies ensuring FERPA and HIPAA compliance. Interfaces should minimize student cognitive load via seamless protections and transparent policies. Access controls should limit data visibility to appropriate stakeholders while maintaining intuitive navigation that does not burden users with complex privacy management tasks.
- *Bias mitigation:* Without careful design and monitoring, AI systems may inadvertently perpetuate existing biases. Institutions should conduct regular audits of AI recommendations to identify inequity patterns or favoritism toward certain groups. Training models on diverse student experiences requires deliberate inclusion of varied demographic data, with audit systems designed to detect when AI responses assume background knowledge that triggers “slow thinking” or perpetuates bias.
- *Maintaining human connection:* Even sophisticated AI systems cannot replace the vital human elements of medical education. Implementation strategies should define clear boundaries and identify which aspects of mentorship remain exclusively human domains. Pathways should be created that refer and connect students to appropriate human mentors when more nuanced guidance is needed. Authentic human relationships need space to develop, which can occur when faculty receive support to effectively integrate AI tools as complements to, rather than replacements for, their essential mentoring roles.

### Implementation Challenges

Two implementation challenges require proactive attention:

- *Privacy and trust:* Students from marginalized backgrounds may have heightened concerns about privacy and data use. Clear policies regarding data collection, usage, and privacy protections are essential for building trust in these systems, particularly among students who may have historical reasons to be wary of institutional technologies [26].
- *Human-AI balance:* While AI can supplement human mentorship, it cannot replace the relational aspects of education that are particularly important for underrepresented students. Implementation should emphasize AI as a complement to, rather than a replacement for, human guidance and support [23].

By addressing these considerations proactively, institutions can leverage AI to democratize hidden curriculum access and create more equitable learning environments for FGLI/URM students.

## Conclusion

The hidden curriculum in medical education creates systematic disadvantages for FGLI/URM students who lack access to generational knowledge and informal networks that privileged peers navigate intuitively. Through the lens of cognitive load theory, navigating hidden curriculum elements imposes substantial extraneous cognitive load on underrepresented students, diverting mental resources from germane processing of formal medical content. This inequity manifests as professional integration lag phases and reduced access to critical opportunities throughout medical training. Generative artificial intelligence offers a transformative solution by reducing extraneous cognitive burden, democratizing access to implicit knowledge, and delivering just-in-time guidance that enables students to redirect cognitive resources toward productive learning.

## Framework for AI-Driven Hidden Curriculum Equity Interventions

Successful implementation requires careful attention to bias mitigation, data privacy, and maintaining essential human connections while embedding AI tools within existing educational frameworks. By systematically addressing the Fast/Slow thinking disparities that perpetuate hidden curriculum inequities, AI-driven interventions can transform medical education into a more equitable system where student success depends on merit and effort rather than background privilege. This framework (Table 1) provides medical institutions with a scalable, evidence-based approach to democratize professional knowledge and create truly inclusive learning environments for all students. By systematically addressing the Fast/Slow thinking disparities across four key implementation components (knowledge access, navigation support, mentorship enhancement, and system integration), AI-driven interventions can transform medical education into a more equitable system where student success depends on merit and effort rather than background privilege.

**Table 1:** Framework for AI-driven hidden curriculum equity interventions in medical education. This implementation matrix demonstrates how artificial intelligence tools can systematically address both fast thinking (automatic, intuitive) and slow thinking (deliberate, analytical) challenges faced by first-generation, low-income (FGLI) and underrepresented minority (URM) students. Each component includes specific AI applications and corresponding implementation strategies designed to reduce cognitive burden and democratize access to implicit professional knowledge

Component	Fast thinking support	Slow thinking support	Implementation strategy
Knowledge Access	Professional language decoders, cultural navigation guides	Strategic study planning, career pathway mapping	NotebookLM workspaces with curated institutional sources
Navigation Support	Parallel curriculum planners, network mappers	Medical education journey mapping, feedback interpretation	LMS integration with contextual awareness
Mentorship Enhancement	Just-in-time templates and examples	Comprehensive residency application strategies	AI-human mentorship pathways with clear boundaries
System Integration	Automated prompting of intuitive questions	Phase-specific modules aligned with curricula	Faculty development programs and ethical frameworks

## Declarations

### Disclaimer

The discussion of Google NotebookLM and other AI platforms reflects the authors’ independent assessment of currently available technologies and does not represent paid promotion or endorsement.
